# Preoperative breast magnetic resonance imaging in patients with ductal carcinoma in situ: a systematic review for the European Commission Initiative on Breast Cancer (ECIBC)

**DOI:** 10.1007/s00330-021-07873-2

**Published:** 2021-05-30

**Authors:** Carlos Canelo-Aybar, Alvaro Taype-Rondan, Jessica Hanae Zafra-Tanaka, David Rigau, Axel Graewingholt, Annette Lebeau, Elsa Pérez Gómez, Paolo Giorgi Rossi, Miranda Langendam, Margarita Posso, Elena Parmelli, Zuleika Saz-Parkinson, Pablo Alonso-Coello

**Affiliations:** 1 CIBER de Epidemiología y Salud Pública (CIBERESP), Madrid, Spain; 2 Iberoamerican Cochrane Centre - Department of Clinical Epidemiology and Public Health, Biomedical Research Institute Sant Pau (IIB Sant Pau), Sant Antonio María Claret 167, 08025 Barcelona, Spain; 3 Universidad San Ignacio de Loyola, Unidad de Investigación para la Generación y Síntesis de Evidencias en Salud, Lima, Peru; 4 CRONICAS Centre of Excellence in Chronic Diseases, Universidad Peruana Cayetano Heredia, Lima, Peru; 5 Radiologie am Theater, Paderborn, Germany; 6 Institute of Pathology, University Medical Center Hamburg-Eppendorf, Hamburg, Germany; 7 University Hospital Dr. Josep Trueta, Girona, Spain; 8 Epidemiology Unit, Azienda USL – IRCCS di Reggio Emilia, Reggio Emilia, Italy; 9 Department of Epidemiology and Data Science, Amsterdam UMC, University of Amsterdam, Amsterdam Public Health Institute, Amsterdam, Netherlands; 10 Department of Epidemiology and Evaluation, IMIM (Hospital del Mar Medical Research Institute), Barcelona, Spain; 11 European Commission, Joint Research Centre (JRC), Via E. Fermi, 2749. TP127, I-21027 Ispra, VA Italy

**Keywords:** Breast cancer, Ductal carcinoma in situ, Magnetic resonance imaging

## Abstract

**Objective:**

To evaluate the impact of preoperative MRI in the management of Ductal carcinoma in situ (DCIS).

**Methods:**

We searched the PubMed, EMBASE and Cochrane Library databases to identify randomised clinical trials (RCTs) or cohort studies assessing the impact of preoperative breast MRI in surgical outcomes, treatment change or loco-regional recurrence. We provided pooled estimates for odds ratios (OR), relative risks (RR) and proportions and assessed the certainty of the evidence using the GRADE approach.

**Results:**

We included 3 RCTs and 23 observational cohorts, corresponding to 20,415 patients. For initial breast-conserving surgery (BCS), the RCTs showed that MRI may result in little to no difference (RR 0.95, 95% CI 0.90 to 1.00) (low certainty); observational studies showed that MRI may have no difference in the odds of re-operation after BCS (OR 0.96; 95% CI 0.36 to 2.61) (low certainty); and uncertain evidence from RCTs suggests little to no difference with respect to total mastectomy rate (RR 0.91; 95% CI 0.65 to 1.27) (very low certainty). We also found that MRI may change the initial treatment plans in 17% (95% CI 12 to 24%) of cases, but with little to no effect on locoregional recurrence (aHR = 1.18; 95% CI 0.79 to 1.76) (very low certainty).

**Conclusion:**

We found evidence of low to very low certainty which may suggest there is no improvement of surgical outcomes with pre-operative MRI assessment of women with DCIS lesions. There is a need for large rigorously conducted RCTs to evaluate the role of preoperative MRI in this population.

**Key Points:**

*• Evidence of low to very low certainty may suggest there is no improvement in surgical outcomes with pre-operative MRI.*

*• There is a need for large rigorously conducted RCTs evaluating the role of preoperative MRI to improve treatment planning for DCIS.*

**Supplementary Information:**

The online version contains supplementary material available at 10.1007/s00330-021-07873-2.

## Introduction

In 2018, globally, an estimated 2 million new cases of breast cancer (BC) were reported [1]. Ductal carcinoma in situ (DCIS) of the breast is the most common form of non-invasive BC, and includes a heterogeneous group of atypical cell proliferation confined within the basement membrane of the ducts [2]. Over the last decades, the detection of DCIS has increased, likely because of the widespread use of screening mammography, accounting for 20 to 25% of newly diagnosed BC in screened populations [3].

Nowadays, breast-conserving surgery (BCS) has been adopted as a treatment option for patients with small, screen-detected lesions [4]. The addition of adjuvant radiation and hormonal therapy after BCS has been shown to reduce the risk of invasive recurrence [4]. However, complete surgical excision is not always possible due to the suboptimal preoperative evaluation of the extent of the lesion by standard imaging (mammography, ultrasound). Therefore, re-operation for positive margins is often required in DCIS, with rates ranging from 17 to 58% [5].

Magnetic resonance imaging (MRI) has a higher sensitivity for BC diagnosis preferentially detecting more aggressive/invasive types [6]. It has been proposed as an additional test after mammography, to improve the assessment of the extent of DCIS during the preoperative planning, providing better identification of candidates for BCS especially in the context of extensive microcalcifications [7]. Despite previous reviews suggesting MRI benefits [8–10], there is still uncertainty as some studies suggest it may overestimate the extent of disease, leading to an increase of unnecessary mastectomies or wider excisions [11, 12].

The European Commission Initiative on Breast Cancer (ECIBC) develops the European Guidelines on Breast Cancer Screening and Diagnosis [13]. This systematic review informed the recommendations of preoperative breast MRI (Prospero register: 42018099453). During the guidelines process [13], the Guidelines Development Group (GDG) made detailed considerations on the evidence about effects, values and preferences, equity, acceptability and feasibility to issue recommendations. We encourage readers to refer to these considerations in the published recommendations on the ECIBC website (https://healthcare-quality.jrc.ec.europa.eu/european-breast-cancer-guidelines/surgical-planning/MRI)

## Methods

### Structured question and outcome prioritisation

The clinical question prioritised by the GDG was “*Should additional MRI vs no additional MRI be used in women with histologically confirmed DCIS for preoperative planning?*”.

Outcomes were prioritised using a 1 to 9 scale as suggested by the Grading of Recommendations Assessment, Development and Evaluation (GRADE) approach (Box 1).

Box 1 Structured clinical question

**Table:** 

Population	Intervention	Comparison	Outcomes
Women with confirmed DCIS on preoperative histology	Preoperative breast MRI	No preoperative breast MRI	• MRI triggered treatment change, as the decision to perform a wider excision, a mastectomy or a bilateral mastectomy when a more conservative approach were originally planned before MRI results • Initial breast-conserving surgery (BCS), a patient not undergoing mastectomy within the initial surgical treatment^a^ • Re-operation after breast-conserving surgery, either a wider local excision or mastectomy after the first surgery • Proportion of positive margins after breast-conserving surgery, absence of clear margins at the pathologic assessment of the specimen after surgical resection • Total mastectomy, the last definitive mastectomy, including initial and additional mastectomy due to re-operation • Disease-free survival (inferred from loco-regional recurrence) • Quality of life

^a^An increase in this outcome is a desirable change as it is a complementary event to initial mastectomy

### Data sources and searches

We searched the MEDLINE (via PubMed, April 2018), EMBASE (via Ovid, April 2018) and CENTRAL (via The Cochrane Library, March 2018) databases using pre-defined algorithms. In addition, we updated our initial search during the first week of January 2021 (Supplementary Table 1), and GDG members were consulted about potential missing studies.

### Study selection

We included randomised controlled trials (RCT) and cohort studies that compared preoperative MRI with no MRI in women with histologically confirmed DCIS. We excluded studies that included women with invasive breast carcinomas (IBC), those that did not provide stratified results for women with DCIS, conference abstracts and articles published in languages other than English.

Initially, two calibrated reviewers (A.T.R. and J.Z.) assessed the eligibility at title and abstract level. In a second step, the two reviewers independently reviewed the full text of all the selected references. Discrepancies were solved by consensus or with the help of a third reviewer (CCA).

### Data extraction and risk of bias assessment

Three reviewers (A.T.R., J.Z., C.C.A.) independently extracted data and assessed the risk of bias using the Cochrane Risk of Bias tool [14] for RCTs and the “Risk Of Bias in Non-randomised Studies of Interventions-I” (ROBINS-I) for observational studies. Before applying the latter tool, we specified relevant confounding variables (i.e. age, family history of BC, tumour size) [15].

### Data analysis

From RCTs, we extracted crude relative risks (RR), and from observational studies, we obtained adjusted odds ratios (aOR) or hazard ratios (aHR) when available. We did not pool the results obtained from both types of designs. Pooled effect sizes were estimated using a random effects model with the Mantel-Haenzel or inverse variance method. To estimate between-study variance and confidence intervals, we used the Paule-Mendel and Q-profile methods. To pool the proportion of MRI-treatment changes, we implemented a generalised linear mixed random model with a logit transformation, and the Clopper-Pearson method to estimate the confidence interval for individual study results.

Heterogeneity between studies was evaluated by visual inspection of forest plots for all outcomes and complemented with the assessment of the *Q* statistic and *I*^2^ parameter for relative effects, as they are not recommended for proportions [16]. The following potential sources of heterogeneity were examined: the extent of microcalcifications, risk of bias, adjusted or crude effect sizes, prospective or retrospective design for observational studies and publication year (post hoc). We performed all analysis in RStudio.

### Certainty of the evidence

We rated the certainty of the evidence separately for RCTs and observational studies for each of the prioritised outcomes using the GRADE approach [17]. For the assessment of the certainty of observational evidence, we started from high certainty, as this is the recommended procedure when the ROBINS-I tool is used [18].

## Results

### Search results

**Fig. 1:**
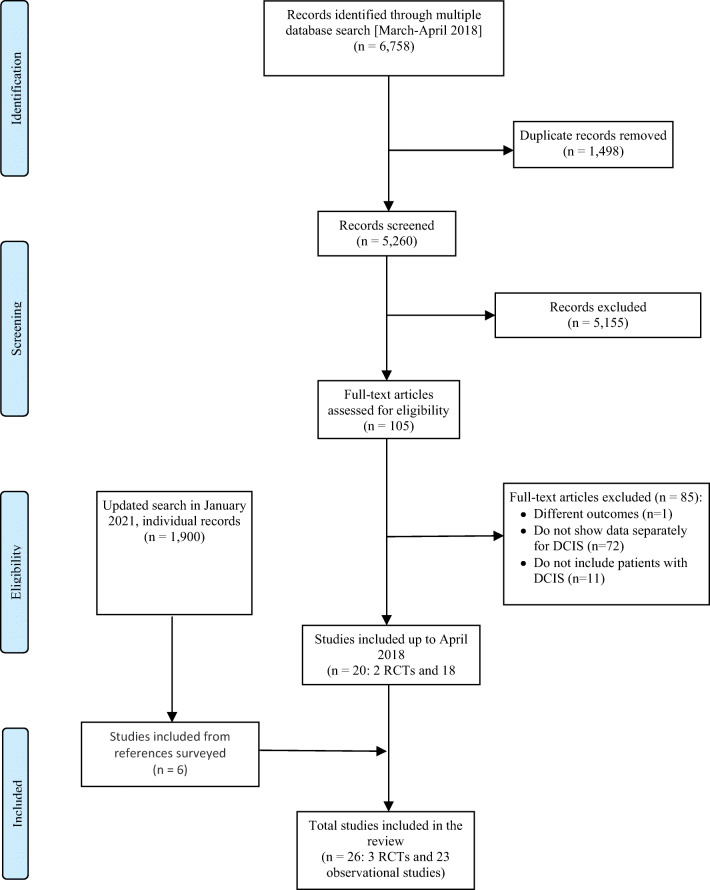
Flowchart of study selection

In the initial search until April 2018, we retrieved 5260 unique citations. Initially, we included a total of 20 studies; this was the original evidence synthesis used to develop the ECIBC recommendations. Finally, after the update search (January 2021), we included six additional studies and the update of a previously included cohort (Fig. 1). Reasons for exclusion are detailed in Supplementary table S2.

### Study characteristics

Three studies were RCTs [19–21], 18 were comparative cohorts [11, 12, 22–36] and five were single arm cohorts [12, 21, 37–39], with a total of 20,415 patients (260 from the RCTs and 20,155 from the observational studies). Most studies were conducted in the USA or in the Netherlands. The mean ages of the recruited patients ranged from 40 to 63 years across all studies. Only one study included all patients with microcalcified lesions, while two studies reported the percentage of microcalcified lesions (range 76 to 99%) (Table 1 and Supplementary table S3).

**Table 1:** Characteristics of the included studies

Author and year (trial)	Country	Design	No. of patients (only DCIS)	Tumour characteristics (microcalcification, size)	Age (years), mean (range)	Intervention (MRI)
Balleyguier (IRCIS) 2019 [21]	France	RCT	MRI: 176 No MRI: 173	Microcalcification: MRI: 98%; No MRI: 99% Mean size (mm): MRI: 10, no MRI: 13	MRI: 56 (31-80) No MRI: 58 (39–80)	1.5 T systems mainly and 3 T systems in 2 centres
Peters (MONET) 2011 [19]	Netherlands	RCT (subgroup analysis)	MRI: 39 No MRI: 41	Microcalcification: NR Mean size (mm): NR	NR	3 T, dedicates phased-array bilateral breast coil
Turnbull (COMICE) 2010 [20]	UK	RCT (subgroup analysis)	MRI: 43 No MRI: 48	Microcalcification: NR Mean size (mm): NR	NR	1.5 T, dedicated breast-surface coils for signal reception, with a few scans done at 1.0 T
Allen 2010 [22]	USA	Retrospective cohort	MRI: 64 No MRI: 35	Microcalcification: NR Mean size (mm): NR	MRI: 60.5 (40–83) No MRI: 64.4 (41–89)	1.5 T using 8 channel breast-surface coil
Besharat 2018 [40]	Iran	One arm retrospective cohort	MRI: 5	Microcalcification: NR Mean size (mm): NR	All patients (DCIS + invasive): 45.5	1.5 T
Davis 2012 [23]	USA	Retrospective cohort, comparing two different time periods	MRI: 154 No MRI: 64	Microcalcification: NR Mean size (mm): NR	NR	1.5-T scanner with use of a dedicated prone eight-channel breast coil
Duygulu 2012 [41]	Turkey	One arm retrospective cohort	MRI: 18	Microcalcification: NR Mean size (mm): NR	NR	1.5 T using a standard breast coil in the prone position
Hajaj 2017 [24]	UK	Retrospective cohort from one hospital, comparing two different time periods	MRI: 70 No MRI: 52	Microcalcification: NR Size range (mm): MRI: 2 to 110; no MRI: 3 to 180	MRI: 63 (31–75) No MRI: (56–82)	1.5-T scanner and a dedicated 8-channel breast coil
Hlubocky 2018 [38]	USA	One arm retrospective cohort in two sites	MRI: 288	Microcalcification: NR Mean size: NR	NR	Initially with 1.5-T magnets; later, all were performed on 3.0 T
Itakura 2011 [11]	USA	Retrospective cohort	MRI: 38; No MRI: 111	Microcalcification: NR Size median (mm): MRI: 16, no MRI: 10	MRI: median: 50 (24–71) No MRI: median: 59 (38–86)	NR
Keymeulen 2019 [33]	Netherlands	Retrospective cohort (population registries)	MRI: 2382 No MRI: 8033	Microcalcification: NR Mean size: NR	MRI: 50–74 years (74%) No MRI: 50–74 years (88%)	NR
Kropcho 2012 [25]	USA	Retrospective cohort from one site	MRI: 62; No MRI: 98	Microcalcification: NR Mean size (mm): MRI: 20.9, no MRI: 27.8	MRI: 55 (35–78) No MRI: 62 (38–93)	1.5-T magnet using a dedicated four-channel in vivo breast coil
Lam 2019 [34]	USA	Retrospective cohort fromone hospital	MRI: 332 No MRI: 41	Microcalcification: NR Mean size: NR	All patients: 55.5	NR
Lamb 2020 [36]	USA	Restrospective cohort from one hospital	MRI: 236 No MRI: 727	Microcalcification: all patients Mean size: NR	MRI: 50.6 ± 8.8 No MRI: 60.2 ± 10	1.5 T or 3 T
Lee 2016 [37], 2020 [34]	Korea	One arm retrospective cohort in one site	MRI: 199	NR	All patients: 50.1 ± 9.4	1.5-T system with a dedicated 4-channel breast coil
Obdeijn 2013 [26]	Netherlands	Retrospective cohort, comparing two different time periods (subgroup analysis)	MRI: 11 No MRI: 27	Microcalcification: NR Mean size (mm): NR	NR	1.5-T system with a dedicated double breast coil
Onega 2017 [27]	USA	Retrospective cohort (Breast Cancer Surveillance Consortium (BCSC))	MRI: 354 No MRI: 2083	Microcalcification: NR Mean size (mm): NR	NR	NR
Pettit 2009 [39]	USA	One-arm retrospective cohort (subgroup analysis)	MRI: 51	Microcalcification: NR Mean size (mm): NR	NR	Siemens 1.5-T Sonata or Espree magnetic resonance imaging unit with dedicated breast coil
Pilewskie 2013 [12]	USA	Prospective cohort from the Lynn Sage Comprehensive Breast Center	MRI: 217; No MRI: 135	Microcalcification: MRI: 75.8 %, no MRI: 93.8 % Mean size cm (range): MRI: 2.1 (0.0, 10.0), no MRI: 1.7 (0.0, 9.0)	MRI: 53 (26–82) No MRI: 60 (36–86)	NR
Pilewskie 2014 [28]	USA	Retrospective cohort from the Memorial Sloan-Kettering Cancer Center (MSKCC)	MRI: 596; No MRI: 1723	Microcalcification: NR Mean size (mm): NR	MRI: 54.0 (26-73) No MRI: 53.5 (25–85)	NR
Shin 2012 [29]	Korea	Retrospective cohort (subgroup analysis)	MRI: 62; No MRI: 25	Microcalcification: NR Mean size (mm): NR	NR	A 1.5-T imager with dedicated double-breast coil was used
So 2018 [30]	USA	Retrospective cohort from one site	MRI: 97; No MRI: 79	Microcalcification: NR Mean size ± SD: MRI: 1.5 ± 1.9, no MRI: 1.6 ± 2.6	MRI: 56.4 No MRI: 63.6	NR
Solin 2008 [42]	USA	Retrospective cohort from one site	MRI: 31 No MRI: 105	Microcalcification: NR Mean size (mm): NR	NR	NR
Vapiwala 2017 [31]	USA	Retrospective cohort (subgroup analysis)	MRI: 31; No MRI: 104	Microcalcification: NR Mean size (mm): NR	Microcalcification: NR Mean size (mm): NR	NR
Vos 2015 [32]	Netherlands	Retrospective cohort, population-based (subgroup analysis—high-grade DCIS)	MRI: 136 No MRI: 478	Microcalcification: NR Mean size (mm): NR	NR	Dynamic contrast-enhanced MRI
Yoon 2020 [35]	Korea	Retrospective cohort from one hospital	MRI: 106 No MRI: 106 (post propensity matching)	Microcalcification: NR Mean size (cm): 3.0 ± 2.4	All patients: 53.5 ± 10 (post propensity matching)	A 1.5-T or 3-T with dedicated double-breast coil was used

*MRI* magnetic resonance imaging, *NR* non-reported, *MC* microcalcifications, *T* tesla, *DCIS* ductal carcinoma in situ

**Table 2:**
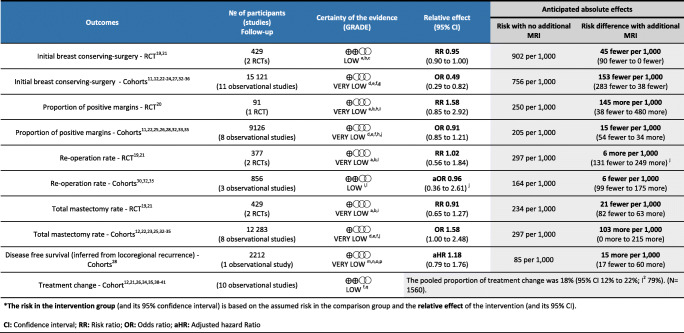
Summary of findings

^a^Risk of bias. The intervention (preoperative MRI) was not feasible to be blinded which led to high risk of performance bias, potentially influencing surgeons’ treatment plans

^b^Risk of bias. There was also a potential risk of imbalance of prognostic factors, due to the inclusion of results from a very small subgroup of participants in some RCTs

^c^Indirectness. Initial BCS was considered an intermediate outcome, as women could have received re-excision or a mastectomy depending on the presence of positive margins in the excised specimen

^d^Risk of bias. In some cohort studies, the comparison was between arms over different periods of time (secular bias)

^e^Risk of bias. Most observational studies reported unadjusted estimates

^f^Inconsistency. Potentially important and unexplained heterogeneity across included studies

^g^Other considerations. Although there is an observed large effect, there is a very serious risk of bias concern and the estimate is imprecise; thus, we did not upgrade the certainty of evidence

^h^Risk of bias. The definition of positive margins was variable across clinical centres potentially introducing misclassification bias

^i^Imprecision. The anticipated absolute effects associated to the intervention go from potential benefit to potential harm

^j^Imprecision. There is imprecision of the anticipated absolute effects with the intervention but it is likely due to heterogeneity across studies

^k^Only estimates from studies reporting adjusted ORs are included as the results were different from those unadjusted

^l^Risk of bias. Both studies reported adjusted estimates, although one study did not include tumour size as a pre-defined confounding variable in the analysis. Additionally, there was no information about the time the MRI exam was requested

^m^Risk of bias. Only patients who received breast-conserving surgery were included. There was potential selection bias as those with more aggressive treatments after MRI were not included. Potential over adjustment in the multivariate analysis as positive margins and number of excisions may be in the casual pathway to disease recurrence

^n^Indirectness. A proportion of patients had breast MRI performed after lumpectomy or at re-excision stage

^o^Indirectness. Serious indirectness as locoregional recurrence is considered a surrogate of disease-free survival

^p^Imprecision. The absolute effect of the intervention ranged from significant benefit to significant harm

^q^Risk of bias. Decision to request breast MRI (after mammography and ultrasound) might be associated to the decision to change the initial plan, independent of MRI results

One RCT was purposely designed to evaluate the value of breast MRI in patients with biopsy-proven DCIS who were scheduled for BCS [21], with almost all lesions presenting microcalcifications and a mean size of 10 mm. Additionally, we included data from small subgroups of patients with DCIS from two RCTs, the MONET trial [19] which randomised patients with a non-palpable BI-RADS 3–5 lesion to receive routine medical care (mammography, ultrasound and lesion sampling) or additional MRI preceding biopsy and the multicentric COMICE trial [20] which recruited patients with biopsy-proven primary BC who had undergone triple assessment, and were scheduled for a wide local excision.

Of the 18 comparative cohorts included [11, 12, 22–34], 16 were retrospective analyses of medical records or population registries [11, 12, 22–27, 29–36], three of them comparing different time periods (pre- and post-implementation of MRI) [23, 24, 26]. Three cohort studies had larger sample sizes, one used the Breast Cancer Surveillance Consortium registry data with 2437 patients with DCIS from 2010 to 2014 [27], another study included data of 2319 patients from a cancer centre in New York (USA) from 1997 to 2010 [28] and the last study used the Netherlands Cancer Registry (NCR) including 10,415 clinical records [33] (Table 1 and Supplementary table S3).

We also included five single MRI-arm cohorts [37–41] and the MRI arm of three comparative cohorts and one RCT [12, 21, 26], where all surgical treatment changes due to the MRI findings were recorded. We did not find studies directly reporting the quality of life of patients receiving pre-operative breast MRI.

### Initial BCS

**Fig. 2:**
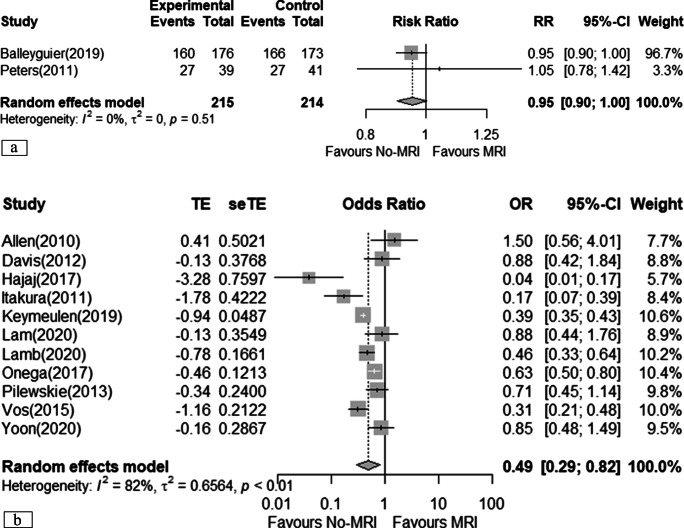
Meta-analyses of initial breast-conserving surgery. **a** Randomised clinical trial; **b** cohort studies (prospective and retrospectives)

Two RCTs, including a total of 429 patients, showed that MRI may result in little to no difference in initial BCS (RR 0.95; 95% CI 0.90 to 1.00) (Fig. 2a) (*low certainty*) [19, 21]. In 11 observational studies [11, 12, 22–24, 27, 32–36], the odds of BCS was lower but the evidence was very uncertain (OR 0.49; 95% CI 0.29 to 0.82) and only two cohort studies reported adjusted OR showing similar results (*very low certainty*) [32, 35] (Fig. 2b).

### Proportion of positive margins (after undergoing BCS)

The results from 91 patients in one RCT suggested that MRI may increase the risk of positive margins in the excised lesion (RR of 1.58; 95% CI 0.88 to 2.92) (*very low certainty*) [20]. However, from observational studies, the pooled analysis including 9126 patients suggested that MRI pre-operative assessment may have little to no difference in this outcome (OR 0.91; 95% CI 0.85 to 1.21) [11, 22, 25, 26, 28, 32, 33, 35] (*very low certainty*). It is noteworthy that the definitions for positive margins across studies were heterogenous (i.e. < 1 mm [11, 27] or < 5 mm [29]) or not clearly described [36], even among the included centres of the only RCT reporting this outcome [20].

### Re-operation rate (after undergoing BCS)

**Fig. 3:**
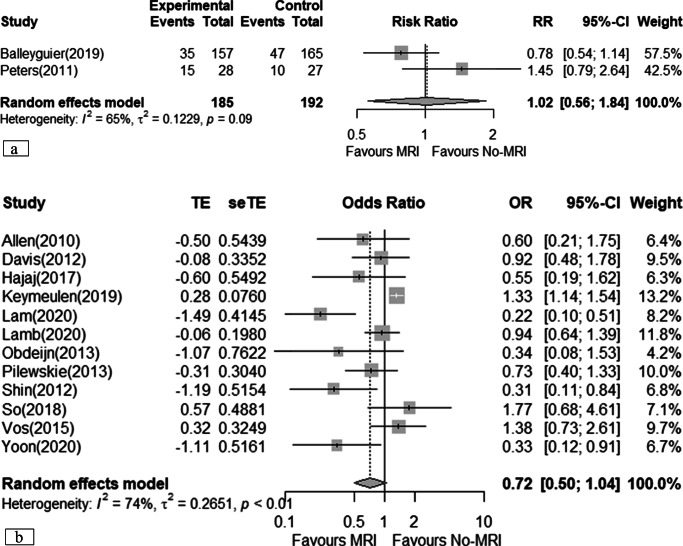
Meta-analyses of re-operation rate. **a** Randomised clinical trial; **b** cohort studies (prospective and retrospectives)

Two RCTs suggested no difference in the risk of having a re-operation after an initial BCS (RR 1.02; 95% CI 0.56 to 1.84), but the evidence was uncertain (*very low certainty*) [19, 21]. The pooled estimate from 12 observational studies suggested a decrease in the risk of re-operation (OR 0.72; 95% CI 0.50 to 1.04) (Fig. 3b) [11, 12, 22–26, 29, 30, 32–36].

The observational studies providing adjusted estimates had an inconsistent effect (OR 0.96; 95% CI 0.36 to 2.61) [30, 32, 35] compared to studies with unadjusted estimates (OR 0.66; 95% CI 0.45 to 0.99). Therefore, only those providing adjusted estimates were included in the evidence profile for observational studies (*low certainty*). One study reported the mean number of re-operations among those patients who received lumpectomy and showed no relevant differences (0.42 vs 0.58, *p* value = 0.31) [11].

### Total mastectomy

**Fig. 4:**
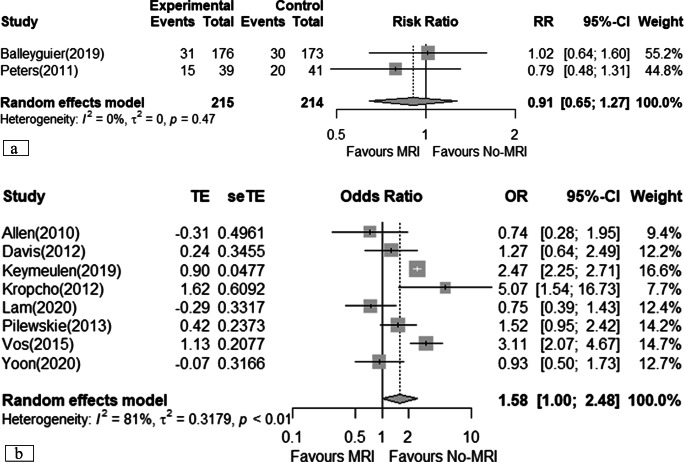
Meta-analyses of total mastectomy. **a** Randomised clinical trial; **b** cohort studies (prospective and retrospectives)

Two RCTs, including 429 patients [19, 21], showed that MRI may result in little to no difference in total mastectomy surgeries (RR 0.91; 95% CI 0.65 to 1.27), equivalent to 21 fewer total mastectomies (95% CI 82 fewer to 63 more) performed per 1000 patients assessed (*very low certainty*) (Table 2). Eight observational studies suggested that MRI may increase the odds of total mastectomies (OR 1.58; 95% CI 1.00 to 2.48) (*very low certainty*) (Fig. 4b) [12, 22, 23, 25, 32–35], with a larger but imprecise effect observed in studies providing adjusted results (aOR 1.74; 95% CI 0.53 to 5.68) [32, 35].

### Disease-free survival (inferred from locoregional recurrence)

One study reported a subgroup of 135 patients with DCIS, over a follow-up of 10 years, and found no differences in disease-free survival between the use or not of breast MRI (4% vs 4%, *p* value = 0.25) [31]. Another study reported similar results at 8 years of follow-up for the rate of any local failure among 136 patients (6% vs 6%, *p* value = 0.58) [42]. Lamb et al reported similar rates of second BC events whether MRI was used or not (12.8% versus 11.5%, *p* = 0.68) [36].

The largest study, which included 2212 patients with a median follow-up of 4.9 years, showed, in an adjusted multivariate analysis (i.e. age, margin status, number of excisions), that MRI may increase the risk of local recurrence (aHR = 1.18; 95% CI 0.79 to 1.76) (*very low certainty*) [28].

### MRI triggered treatment change

Eight cohorts [12, 26, 33, 35, 37–41] and the intervention arm of one RCT [21] informed this outcome. Our pooled estimation showed that 17% (95% CI 12 to 24%) of the initial surgical decisions may change to a more extensive resection or mastectomy when breast MRI is used (*low certainty*) (Supplementary Figure 2). There was important heterogeneity, with the five studies with a larger sample size [12, 21, 37–39] reporting between 9 and 18% and those with a smaller sample size reporting much higher proportions (39% [41] to 50% [26]).

### Subgroup and sensitivity analysis

One RCT and 11 observational studies reported the age of participants which was similar across studies. We were not able to perform subgroup analysis on the extent of microcalcifications as most studies did not provide detailed data, but we included a post hoc analysis comparing those studies that reported the proportion of patients with microcalcification lesions with those that did not.

As described above (see “Re-operation rate (after undergoing BCS)”), we found different estimates among observational studies reporting adjusted versus crude estimates for re-operations but with overlapping confidence intervals (test for subgroup differences *p* value = 0.499). Other subgroup analyses, including publication year, did not show meaningful results (Supplementary table S4).

### Risk of bias and certainty of the evidence

The included RCTs did not blind participants nor the clinical personnel leading to a potential risk of performance bias as treatment decisions might have been influenced by the knowledge of the allocation arm. The assessment of surgical outcomes is less likely to be biased due to unblinded assessment. However, the evaluation of positive margins in the surgical excised specimens might be at higher risk of bias as described elsewhere [43].

In addition, two of the three RCTs might be subject to an imbalance of prognosis factors, as we included results for small subgroups of the originally allocated patients [19, 20]. The probability of important imbalance for a single prognostic factor is higher in RCTs with less than 100 participants [44, 45].

For observational evidence, our main concern was risk of bias, as only three studies reported adjusted estimates [28, 30, 32]. Confounding variables (i.e. age, lesion size on mammography) might be associated to requesting breast MRI and to performing more aggressive surgical treatments. Most observational studies were retrospective and potentially subject to loss of follow-up or misrecorded data. Three studies compared two different time periods which may lead to bias related to different standards of care due to the progress in treatment quality over time [23, 24, 26].

For the assessment of the certainty of evidence, we rated down for risk of bias for all outcomes, for inconsistency in most estimates informed by observational studies, and for imprecision for locoregional recurrence, positive margins estimated from RCTs, and for re-operation estimates from both RCTs and observational evidence (Table 2 and Supplementary table S5).

## Discussion

### Main findings

Our review suggests that pre-operative breast MRI for DICS lesions may have no meaningful impact on surgical outcomes or on the risk of local recurrence. RCTs showed that MRI had little to no effect on initial BCS or total mastectomies, a finding that was consistent also with the results from observational studies. For re-operations, there is also uncertainty; the IRCIS trial designed to include only DCIS patients suggested a reduction but with confidence intervals including the opposite effect [21], while the MONET trial showed an increase in a small DCIS subgroup [19]. Among observational studies, Yoon et al, using propensity score matching, suggested a reduction in re-operations [35]. However, another two observational studies providing adjusted estimates did not find a benefit with MRI [30, 32].

The included RCTs had several limitations [46]. The IRCIS trial recruited patients eligible for BCS after mammography, ultrasound and percutaneous biopsy which might have biased mastectomy effect estimates against MRI. This study might also be underpowered as there were meaningful differences in the results between the intention to treat and per protocol analysis [21]. The MONET [19] and COMICE [20] trials randomised women with non-palpable or BIRADS 3–5 lesions respectively, while women with DCIS were only a small fraction of them. Moreover, these RCTs reported difficulties in the acquisition of MIR images at 3 T before randomisation [19], underwent MRI prior to biopsy which is not considered standard of care [19] and guided biopsies to verify MRI findings were not available in all cases [20].

### Our results in the context of previous research

A systematic review including a lower number of studies than our review showed that preoperative breast MRI in patients with DCIS was not associated with an improvement in surgical outcomes [47]. Similar results were also observed in patients with invasive cancer [48–51], with a systematic review showing that MRI increased mastectomy rates [50] and one individual person-data meta-analysis reporting that MRI does not reduce the risk of local recurrence or distant recurrence in these patients [51].

One potential explanation for the lack of benefit on surgical outcomes might be a limited specificity of MRI in patients with DCIS, and variable positive predictive value from 25 to 84% [52]. Previous data has shown that MRI, compared to histopathology, tends to overestimate the size of pure DCIS lesions and has moderate correlation with pathologically measured tumour size (*r* = 0.74) [53, 54]. This limitation could impact on having more aggressive treatments than needed, but not on the number of re-excisions. Furthermore, it should have only hampered older studies where biopsies were not performed in all new lesions identified by MRI and patients frequently went directly to reassessment of the surgical treatment plan.

An important factor is to ensure MRI images were adequately acquired. The ACR Breast MRI Accreditation Program began accrediting facilities in 2010 [55], requiring adequate magnetic field strength (1 T or higher) and gradient, bilateral breast coil enabling prone positioning and good fat suppression [55]. Older studies might be prone to unstandardised procedures as accreditation was not uniformly implemented. Currently, most facilities perform 1.5 T MRI, but the use of 3-T magnetic field has increased [56]. As some studies suggest, despite some technical limitations, 3 T could provide higher correlation with DCIS pathology size compared to 1.5 T, therefore obtaining higher image quality scores and better differential diagnosis of enhancing lesions [57]. Another study, including 20 DCIS lesions, found that size correlation between MRI images and pathology was higher with 3 T [58]. Most studies in our review used 1.5 T; thus, further studies are needed to assess the clinical impact of 3 T.

To provide an optimal accuracy, breast MRI should ideally provide high spatial and temporal resolution [59]; however, conventional MRI methods cannot deliver both and usually prioritise spatial resolution [59]. To solve this issue, recent technical advancements have focused on accelerating data acquisition. Morrison et al described a method that provided six-times faster effective temporal resolution and the same high spatial resolution of standard MRI [60]. Goto et al improved the temporal resolution with preservation of spatial resolution using ultra-fast DCE-MRI to differentiate malignant and benign lesions [61]. As new techniques are developed, the performance of MRI in the preoperative setting could improve.

## Limitations and strengths

We included only English language articles although we included a larger number of studies than previous reviews. We could not explore the effect of MRI in some relevant patient subgroups as this data was not available. Our results are hampered by the low to very low certainty of the evidence found for the included outcomes.

Our review has several strengths. We included outcomes that were of interest for women, clinicians and policy makers when facing the decision of implementing or recommending preoperative MRI, and used rigorous methods including the GRADE approach to rate the certainty of the evidence.

## Implications for practice and research

Patients and clinicians should be aware that although breast MRI for pre-operative assessment may improve the morphological description of DCIS lesions, we did not find evidence to suggest an improvement of surgical outcomes. In fact, observational evidence, although of very low certainty, may suggest that preoperative MRI could lead to more aggressive treatments. Also, MRI-guided biopsy to confirm new lesions or important expansions of the detected lesions is not always feasible or accessible. From a health system perspective, preoperative MRI implies greater resource use [62, 63], and given the uncertain potential benefits, the use of MRI has probably limited interest [64].

Given the uncertain evidence, there is a need for conducting well-powered RCTs assessing the role of preoperative MRI during treatment planning in patients with DCIS lesions incorporating new advancements in MRI imaging acquisition, securing the availability of experienced imaging readers and biopsy confirmation of new lesions.

## Supplementary Information

ESM 1 (DOCX 517 kb)

## Abbreviations

aOR: Adjusted odds ratios
BCS: Breast-conserving-surgery
DCIS: Ductal carcinoma in situ
ECIBC: European Commission Initiative on Breast Cancer
GDG: Guidelines Development Group
IBC: Invasive breast carcinomas
MRI: Magnetic resonance imaging
OR: Odds ratios
PICO: Population, Intervention, Comparison and Outcomes
RCTs: Randomised clinical trials
ROBINS-I: Risk Of Bias in Non-randomised Studies of Interventions-I
RR: Relative risks
SoF: Summary of findings

## Declarations

### Guarantor

The scientific guarantor of this publication is Carlos Canelo-Aybar.

### Statistics and biometry

No complex statistical methods were necessary for this paper.

### Study subjects or cohorts overlap

The studies included in this systematic review were all already published elsewhere. Their references and characteristics are reported within the manuscript.

### Methodology

• retrospective (systematic review of already published studies)

• performed at one institution
