# Correction to: Preoperative breast magnetic resonance imaging in patients with ductal carcinoma in situ: a systematic review for the European Commission Initiative on Breast Cancer (ECIBC)

**DOI:** 10.1007/s00330-021-08489-2

**Published:** 2022-01-07

**Authors:** Carlos Canelo-Aybar, Alvaro Taype-Rondan, Jessica Hanae Zafra-Tanaka, David Rigau, Axel Graewingholt, Annette Lebeau, Elsa Pérez Gómez, Paolo Giorgi Rossi, Miranda Langendam, Margarita Posso, Elena Parmelli, Zuleika Saz-Parkinson, Pablo Alonso-Coello

**Affiliations:** 1 CIBER de Epidemiología y Salud Pública (CIBERESP), Madrid, Spain; 2 Iberoamerican Cochrane Centre - Department of Clinical Epidemiology and Public Health, Biomedical Research Institute Sant Pau (IIB Sant Pau), Sant Antonio María Claret 167, 08025 Barcelona, Spain; 3 Universidad San Ignacio de Loyola, Unidad de Investigación para la Generación y Síntesis de Evidencias en Salud, Lima, Peru; 4 CRONICAS Centre of Excellence in Chronic Diseases, Universidad Peruana Cayetano Heredia, Lima, Peru; 5 Radiologie am Theater, Paderborn, Germany; 6 Institute of Pathology, University Medical Center Hamburg-Eppendorf, Hamburg, Germany; 7 University Hospital Dr. Josep Trueta, Girona, Spain; 8 Epidemiology Unit, Azienda USL – IRCCS di Reggio Emilia, Reggio Emilia, Italy; 9 Department of Epidemiology and Data Science, Amsterdam UMC, University of Amsterdam, Amsterdam Public Health Institute, Amsterdam, Netherlands; 10 Department of Epidemiology and Evaluation, IMIM (Hospital del Mar Medical Research Institute), Barcelona, Spain; 11 European Commission, Joint Research Centre (JRC), Via E. Fermi, 2749. TP127, I-21027 Ispra, VA Italy

**Correction to: Eur Radiol (2021) 31:5880–5893**

**https://doi.org/10.1007/s00330-021-07873-2**

In this article the author name Miranda Langendam was incorrectly written as Miranda Langedam. The corrected author list is given above. The original article has been corrected.

