# Supplementary material for: Preoperative breast magnetic resonance imaging in patients with ductal carcinoma in situ: a systematic review for the European Commission Initiative on Breast Cancer (ECIBC)
Source: Eur Radiol. 2021 May 30;31(8):5880–93. doi: 10.1007/s00330-021-07873-2 (PMC8270803; doi:10.1007/s00330-021-07873-2)
Supplement: Supplementary file 1 — (DOCX 517 kb) [file 330_2021_7873_MOESM1_ESM.docx]

# Supplementary material

## Table S1. Search strategy

| MEDLINE  PubMed  04/12/2018  (later updated on 01/01/2021) | #1 "Magnetic Resonance Imaging"[Mesh] (463836)  #2 MRI[Title/Abstract] (259916)  #3 magnetic resonance[Title] (85101)  #4 #1 OR #2 OR #3 (576666)  #5 “Breast Neoplasms”[Majr] (252939)  #7 DCIS[Title/Abstract] (5233)  #7 “Breast Carcinoma In Situ”[Mesh] (10434)  #8 “Carcinoma, Ductal, Breast”[Mesh] (15958)  #9 “Carcinoma, Intraductal, Noninfiltrating”[Mesh] (10299)  #10 breast[ti] (282432)  #11 ductal carcinoma in situ[tiab] (7051)  #12 #5 OR #6 OR #7 OR #8 OR #9 OR #10 OR #11 (333694)  #13 “Preoperative Care”[Mesh] (70265)  #14 preoperative[Title/Abstract] (285477)  #15 pre operative[Title/Abstract] (28254)  #16 perioperative[Title/Abstract] (104579)  #17 peri operative[Title/Abstract] (7067)  #18 surgical[Title/Abstract] (1004625)  #19 surgery[Title] (372890)  #20 surgery[sh] (2084993)  #21 size[Title/Abstract] (960312)  #22 #13 OR #14 OR #15 OR #16 OR #17 OR #18 OR #19 OR #20 OR #21 (3674788)  #23 #4 AND #12 AND #22 (3770) |
| --- | --- |
| The Cochrane Library  04/13/2018  (later updated on 01/01/2021) | #1 MeSH descriptor: [Magnetic Resonance Imaging] (7799)  #2 "magnetic resonance":ti,ab,kw (27545)  #3 MRI:ti,ab,kw (24037)  #4 #1 OR #2 OR #3 (36401)  #5 MeSH descriptor: [Breast Neoplasms] this term only (13042)  #6 DCIS:ti,ab,kw (477)  #7 MeSH descriptor: [Breast Carcinoma In Situ] (206)  #8 MeSH descriptor: [Carcinoma, Ductal, Breast] (351)  #9 MeSH descriptor: [Carcinoma, Intraductal, Noninfiltrating] (184)  #10 "breast":ti (31301)  #11 "ductal carcinoma in situ":ti,ab,kw (476)  #12 #5 OR #6 OR #7 OR #8 OR #9 OR #10 OR #11 (33804)  #13 MeSH descriptor: [Preoperative Care] (5942)  #14 preoperative:ti,ab,kw (36325)  #15 "pre operative":ti,ab,kw (5004)  #16 perioperative:ti,ab,kw (19217)  #17 "peri operative":ti,ab,kw (1994)  #18 surgical:ti,ab,kw (98337)  #19 surgery:ti (55128)  #20 MeSH descriptor: [General Surgery] (353)  #21 size:ti,ab,kw (74823)  #22 #13 OR #14 OR #15 OR #16 OR #17 OR #18 OR #19 OR #20 OR #21  (224892)  #23 #4 AND #12 AND #22 (311) |
| EMBASE  Ovid Embase  04/13/2018  (later updated on 01/01/2021) | #1 exp nuclear magnetic resonance imaging/ (996485)  #2 MRI.ab,ti. (432814)  #3 magnetic resonance.ti. (101789)  #4 #1 OR #2 OR #3 (1048412)  #5 *breast tumor/ (47447)  #6 DCIS.ab,ti. (9647)  #7 exp breast carcinoma in situ/ (17454)  #8 exp Paget nipple disease/ (7592)  #9 exp intraductal carcinoma/ (16962)  #10 ductal carcinoma in situ.ab,ti (10277)  #11 ductal cancer.ab,ti. (665)  #12 breast cancer.ti. (251473)  #13 #5 OR #6 OR #7 OR #8 OR #9 OR #10 OR #11 OR #12 (295617)  #14 exp preoperative care/ (38108)  #15 preoperative.ab,ti. (348640)  #16 pre operative.ab,ti. (54196)  #17 perioperative.ab,ti. (141474)  #18 peri operative.ab,ti. (15331)  #19 surgical.ab,ti. (1276983)  #20 surgery.ab,ti. (1599130)  #22 size.ab,ti (1202238)  #23 #14 OR #15 OR #16 OR #17 OR #18 OR #19 OR #20 OR #21 OR #22 (3579423)  #24 #4 AND #13 AND #23 (4656) |

## Table S2. List of excluded studies and reasons for exclusion

| **Author** | **Year** | **Reason for exclusion** |
| --- | --- | --- |
|  | | |
| 1. Abe | 2012 | Does not show data separately for DCIS |
| 1. Adkisson | 2012 | Does not show data separately for DCIS |
| 1. Adkisson | 2011 | Does not show data separately for DCIS |
| 1. Angarita | 2010 | Does not show data separately for DCIS |
| 1. Ayoola | 2011 | Does not show data separately for DCIS |
| 1. Bae | 2016 | Does not include patients with DCIS |
| 1. Baltzer | 2010 | Does not show data separately for DCIS |
| 1. Behrendt | 2014 | Does not include patients with DCIS |
| 1. Bilimoria | 2007 | Does not show data separately for DCIS |
| 1. Bitencourt | 2015 | Does not include patients with DCIS |
| 1. Breslin | 2013 | Does not show data separately for DCIS |
| 1. Burnside | 2016 | Does not include patients with DCIS |
| 1. Buxant | 2007 | Does not show data separately for DCIS |
| 1. Byon | 2014 | Does not show data separately for DCIS |
| 1. Carreira Gomez | 2015 | Does not show data separately for DCIS |
| 1. Chandwani | 2014 | Does not show data separately for DCIS |
| 1. Chang | 2009 | Does not show data separately for DCIS |
| 1. Cheung | 2015 | Does not show data separately for DCIS |
| 1. Ciocchetti | 2009 | Does not show data separately for DCIS |
| 1. Davidson | 1997 | Does not show data separately for DCIS |
| 1. Della Corte | 2014 | Does not show data separately for DCIS |
| 1. Dietzel | 2011 | Does not show data separately for DCIS |
| 1. El Khouli | 2014 | Does not show data separately for DCIS |
| 1. Elshof | 2010 | Does not show data separately for DCIS |
| 1. Francescutti | 2002 | Different outcomes |
| 1. Fu | 2015 | Does not show data separately for DCIS |
| 1. Giezen | 2012 | Does not show data separately for DCIS |
| 1. Gilbert | 2014 | Does not show data separately for DCIS |
| 1. Grady | 2012 | Does not show data separately for DCIS |
| 1. Grady | 2010 | Does not show data separately for DCIS |
| 1. Guilfoyle | 2014 | Does not include patients with DCIS |
| 1. Gurdal | 2013 | Does not show data separately for DCIS |
| 1. Han | 2012 | Does not include patients with DCIS |
| 1. Hawley | 2014 | Does not show data separately for DCIS |
| 1. Karamchandani | 2015 | Does not show data separately for DCIS |
| 1. Katipamula | 2009 | Does not show data separately for DCIS |
| 1. Killelea | 2013 | Does not show data separately for DCIS |
| 1. Kim | 2014 | Does not show data separately for DCIS |
| 1. Komatsu | 2004 | Does not show data separately for DCIS |
| 1. Kuroki | 2002 | Does not show data separately for DCIS |
| 1. Lau | 2011 | Does not show data separately for DCIS |
| 1. Levrini | 2011 | Does not show data separately for DCIS |
| 1. Lim | 2010 | Does not show data separately for DCIS |
| 1. Lobbes | 2015 | Does not show data separately for DCIS |
| 1. Mallory | 2015 | Does not show data separately for DCIS |
| 1. Mazilu | 2013 | Does not show data separately for DCIS |
| 1. Mazurowski | 2015 | Does not include patients with DCIS |
| 1. Mennella | 2015 | Does not show data separately for DCIS |
| 1. Moon | 2013 | Does not show data separately for DCIS |
| 1. Moy | 2010 | Does not show data separately for DCIS |
| 1. Orzalesi | 2016 | Does not show data separately for DCIS |
| 1. Ozanne | 2017 | Does not show data separately for DCIS |
| 1. Paakko | 2005 | Does not show data separately for DCIS |
| 1. Pallone | 2014 | Does not include patients with DCIS |
| 1. Park | 2015 | Does not show data separately for DCIS |
| 1. Parsyan | 2016 | Does not include patients with DCIS |
| 1. Patel | 2015 | Does not show data separately for DCIS |
| 1. Pediconi | 2012 | Does not show data separately for DCIS |
| 1. Petrillo | 2013 | Does not show data separately for DCIS |
| 1. Ponzone | 2015 | Does not show data separately for DCIS |
| 1. Sardanelli | 2010 | Does not show data separately for DCIS |
| 1. Schell | 2009 | Does not show data separately for DCIS |
| 1. Schilling | 2011 | Does not show data separately for DCIS |
| 1. Schmitz | 2015 | Does not include patients with DCIS |
| 1. Schnall | 2006 | Does not show data separately for DCIS |
| 1. Scomersi | 2010 | Does not show data separately for DCIS |
| 1. Seely | 2016 | Does not show data separately for DCIS |
| 1. Siegmann | 2009 | Does not show data separately for DCIS |
| 1. Siegmann | 2010 | Does not show data separately for DCIS |
| 1. Steen | 2013 | Does not show data separately for DCIS |
| 1. Sung | 2013 | Does not include patients with DCIS |
| 1. Sung | 2014 | Does not show data separately for DCIS |
| 1. Taneja | 2012 | Does not show data separately for DCIS |
| 1. Thomassin-Naggara | 2013 | Does not show data separately for DCIS |
| 1. Uematsu | 2011 | Does not show data separately for DCIS |
| 1. Van Goethem | 2004 | Does not show data separately for DCIS |
| 1. Wang | 2016 | Does not show data separately for DCIS |
| 1. Weber | 2012 | Does not show data separately for DCIS |
| 1. Xia | 2014 | Does not show data separately for DCIS |
| 1. Yabuuchi | 2006 | Does not show data separately for DCIS |
| 1. Yabuuchi | 2008 | Does not show data separately for DCIS |
| 1. Yamashiro | 2009 | Does not show data separately for DCIS |
| 1. Yoo | 2013 | Does not show data separately for DCIS |
| 1. Young | 2012 | Does not show data separately for DCIS |
| 1. Zeggelink | 2003 | Does not show data separately for DCIS |
| 1. Onega | 2018 | Does not show data separately for DCIS |
| 1. Brück | 2018 | Does not include patients with DCIS |
| 1. Shi-Yi | 2018 | Does not include patients with DCIS |
| 1. Besharat | 2018 | Does not show data separately for DCIS |
| 1. Gray | 2018 | Does not include patients with DCIS |
| 1. Kendal | 2020 | Cost-effectiveness analysis |
| 1. Mariscoti | 2019 | Intervention arm was DBT plus MRI |
| 1. Zeng | 2020 | No stratified analysis for DCIS patients |

## Table S3. Characteristics of included studies

| **Author (year)** | **Country, period** | **Design** | **Inclusion/exclusion criteria** | **Nº of patients with DCIS** | **Tumor characteristics** | **Age (yrs), mean (range)** | **Intervention (MRI)** | **Outcomes** |
| --- | --- | --- | --- | --- | --- | --- | --- | --- |
| **Randomised Controlled Trials** | | | | | | | | |
| Balleyguier 2019 | France, March 2010 to June 2014 | RCT in 10 hospitals | Inclusion: Women between 18 and 80 years of age with a biopsy-proven limited breast DCIS, corresponding to a unifocal microcalcification cluster or a mass less than 30 mm, and scheduled for BCS  Exclusion: patients with invasive carcinoma; with microcalcification foci inaccessible to biopsy; with bilateral lesions; with known contraindications to MRI; who refused surgery, including mastectomy if necessary; with a history of ipsilateral breast cancer; who were at high risk for breast cancer (BRCA1 or 2 mutations); and who were pregnant or breastfeeding | 349 (MRI: 176, No-MRI: 173) | - MC: MRI: 98%; No-MRI: 99% - Size mean (mm): MRI: 10, No-MRI: 13 | MRI: 56 (31-80)  No-MRI: 58 (39-80) | MRI was performed  mainly on 1.5T systems and on 3T systems in two centers | - Initial mastectomy - Initial BCS - Re- operation rate - Total mastectomy rate |
| Peters 2011 | Netherlands | RCT (subgroup analysis of the MONET trial) in 4 hospitals | Inclusion: patients with nonpalpable suspicious breast lesions (BIRADS category 3, 4 or 5) detected on mammography or breast ultrasound  Exclusion: patients with: palpable lesions, age below 18 years, breast surgery or radiation therapy less than nine months prior to inclusion, pregnancy or lactation, obesity (>130 kg), claustrophobia, inability to maintain prone position for one hour, or other general contra-indications for MRI (e.g. pacemaker, other metal implants).* | 80 (MRI: 39; no-MRI: 41) | Data was not reported for DCIS patients.  For all the 456 included patients:   - Microcalcification: MRI: 60%; No-MRI: 58% - Size median (mm): MRI: 15.0; no-MRI: 15.1 | Data was not reported for DCIS patients.  For all the 456 included patients:   - MRI: 55.1 - No MRI: 56.1 | 3-T, dedicates phased-array bilateral breast coil. | - Initial mastectomy - Initial BCS - Re-operation rate - Total mastectomy rate |
| Turnbull 2010 | United Kingdom | RCT (multiple centers) in 45 UK centres | Inclusion: 18 years or older with biopsy-proven primary breast cancer scheduled for wide local excision  Exclusion: patients that are medically unstable; had contraindications to MRI; had undergone chemotherapy or hormonal therapy for contralateral breast in previous 12 months, or chemotherapy to any site before breast surgery; had previous surgery or radiotherapy for cancer to the ipsilateral breast or previous surgery to the ipsilateral breast within the past 4 months for benign breast disease; had a history of serious breast trauma within the past 3 months; were pregnant or breastfeeding; or had a disability preventing MRI in a prone position. | 91 (MRI: 43; no-MRI: 48) | Data was not reported for DCIS patients.  For all the 1623 included patients:   - Microcalcification: MRI: 20.6%, No-MRI: 21.8% - Size median (mm): MRI: 15.0, no-MRI: 15.0 | Data was not reported for DCIS patients.  For all the 1623 included patients:   - MRI: 56.4 - No MRI: 56.6 | 1.5T, dedicated bilateral breast-surface coils for signal reception, with a few scans done at 1.0T. | - Positive margin status |
| **Observational studies:** | | | | | | | | |
| Allen 2010 | United States  2007 | Concurrent retrospective cohort.  Retrospective chart review from 3 hospitals. | Inclusion: Patients with new diagnosis of DCIS who underwent minimally invasive biopsy  Exclusion: NR | 99 (MRI: 64; no-MRI: 35) | NR | - Total: 61.9 (40 to 89) - MRI: 60.5 (40 – 83) - No-MRI: 64.4 (41 – 89) | 1.5-T GE, using 8 channel breast-surface coil (VIBRANT software) | - Initial mastectomy - Initial BCS - Positive margin status - Re- operation rate - Total mastectomy rate |
| Besharat 2018 | Iran 2014-2015 | One arm retrospective cohort in one site | Inclusion: Breast cancer pathologically confirmed.  Exclusion: NR | 5 (all patients had MRI) | NR | All patients with or without DCIS: 45.6 years | 1.5 T | - Treatment change |
| Davis 2012 | United States 2007-2011 | Non-concurrent retrospective cohort (women who underwent surgical treatment for DCIS at Dartmouth Hitchcock Medical Center)  MRI: 2009-2011  No-MRI: 2007-2008 | Inclusion: DCIS confirmed by fine-needle or preoperative core biopsy.  Exclusion: Women treated with excisional biopsy, partial mastectomy at an outside institution, women with recurrent disease, women diagnosed with breast cancer based on MRI detection in the context of high-risk screening, and women diagnosed with invasive cancer in the ipsilateral breast on final pathology. | 218 (MRI: 154; no-MRI: 64) | NR | NR | 1.5-T MR scanner with use of a dedicated prone eight-channel breast coil. | - Initial mastectomy - Initial BCS - Re- operation rate - Total mastectomy rate |
| Duygulu 2012 | Turkey 2006-2008 | One arm retrospective cohort | Inclusion: Patients with cytologically or histopathologically proven breast cancer, with clinical stage of 0, 1, or 2 (T2) according to the physical examination, MMG, and US findings (usual imaging protocol), who underwent pre-operative MRI examination prior to or following the biopsy, had histopathological confirmation of additional findings with the MRI, and revised treatment planning based on the true-positive findings with the MRI.  Exclusion: Cases in which multifocality or multicentricity were observed in multiple quadrants according to the MMG and US findings | 18 (all patients had MRI) | NR | Data was not reported for DCIS patients.  For all the 68 included patients: 40.2 (26 – 60) | 1.5-T. The conventional breast MRI protocol was performed using a standard breast coil in the prone position. | - Treatment change |
| Hajaj 2017 | United Kingdom 2009-2015 | Non-concurrent retrospective cohort in one hospital  No-MRI: March 2009 to March 2011  MRI: April 2011 to December 2015 | Inclusion: women with high grade DCIS.   - For MRI group: patients with a proven HG DCIS on their biopsy, with initial imaging (mammography/ ultrasound) and biopsy followed by discussion at the multidisciplinary team meeting (MDT). - For non-MRI group: patients who had mammographic microcalcification on their mammography (BIRADS 3/4/5) with pure HG DCIS on their biopsy, only had conventional imaging with mammography /ultrasound prior to discussing surgical management.   Exclusion: None | 122 (MRI: 70; No-MRI: 52) | - Microcalcification: NR - Size range (mm): MRI: 2 to 110; no-MRI: 3 to 180 | - MRI: 63 (31-75) - No-MRI: (56-82) | 1.5 Phillips Achieva scanner and a dedicated 8 channels breast coil. | - Initial mastectomy - Initial BCS - Re- operation rate |
| Hlubocky 2018 | United States 2004-2009 | One arm retrospective cohort in two sites | Inclusion: newly diagnosed breast cancer patients who underwent preoperative breast MRI during the study period  Exclusion: None | 288 (all patients had a MRI) | Data was not reported for DCIS patients.  For all the 1352 included patients:   - Size mean: 21.2 mm (range 1-120 mm). - Microcalcification: NR | Data was not reported for DCIS patients.  For all the 1352 included patients: 54 (26 – 88) | At the beginning of the study period, all breast MRIs were performed on 1.5 Tesla magnets, and by the end they were all performed on 3.0 Tesla magnets. | - Treatment change |
| Itakura 2011 | United States 2000-2007 | Concurrent retrospective cohort at the University of California, San Francisco | Inclusion: patients with DCIS without invasive cancer.  Exclusion: women who received neoadjuvant treatment for DCIS, bilateral DCIS. | 149 (MRI: 38; No-MRI: 111) | - Size median (mm): MRI: 16, no-MRI: 10 - Microcalcification: NR | - MRI: median: 50 (24-71) - No MRI: median: 59 (38-86) | NR | - Initial BCS - Positive margin status - Re- operation rate |
| Keymeulen 2019 | Netherlands  2011 to 2015 | Population based study based on the Netherlands | Inclusion: all women aged less than 75 years and treated with surgery for pure DCIS. MRI was considered in patients with high-grade DCIS preferring BCS, unclear tumour size or if there was suspicion of microinvasion based on the preoperative biopsy.  Exclusion: NR | 10415 (MRI: 2382; No-MRI: 8033) | - Size mean (mm): NR - Microcalcification: NR | - MRI: 50 to 74 years (74%)  - No-MRI: 50 to 74 years (88%) | NR | - Initial BCS - Re- operation rate - Total mastectomy |
| Kropcho 2012 | United States 2002-2009 | Concurrent retrospective cohort in one site | Inclusion: Female patients over 18 years of age, diagnosed with DCIS (core biopsy), who underwent BCS  Exclusion: invasive or microinvasive disease, who did not undergo operation at the Institute, or whose initial surgical procedure was mastectomy | 160 (MRI: 62; no-MRI: 98) | - Size mean (mm): MRI: 20.9, no-MRI: 27.8 - Microcalcification: NR | - Total: 60 (35 – 93) - MRI: 55 (35–78) - No MRI: 62 (38–93) | 1.5 Tesla magnet using a dedicated four-channel In Vivo breast coil. | - Positive margin status - Total mastectomy rate |
| Lam 2019 | Unitaed States  2004 to 2013 | Concurrent retrospective | Inclusion: women ≥18 years old without a personal history of ipsilateral breast cancer diagnosed with pure DCIS on CNB. Surgeries were performed by surgeons specialized in breast surgical oncology with the initial approach determined through a shared decision making process.  Exclusion: NR | 373 (MRI: 332; no-MRI: 41) | - Size mean (mm): NR - Macrocalcification: NR | Mean age (all patients): 55.5 +/- 11.3 years | NR | - Initial BCS - Positive margin - Re- operation rate - Total mastectomy |
| Lamb 2020 | United States 2007 - 2016 | Retrospective cohort | Inclusion: (1) presented for screening mammography, (2) were found to have suspicious calcifications, and (3) underwent vacuum assisted breast biopsy yielding pure DCIS  Exclusion: NR | 963 (MRI: 236; no-MRI: 727) | - Microcalcification: all patients - Size (mean): NR | Mean age: MRI 50.6 (8.8); no-MRI 60.2 (10) | 1.5 T or 3 T | - Initial BCS - Re- operation rate |
| Lee 2016, 2020* | Korea 2006 - 2014 | One arm retrospective cohort in Kyungpook National University Hospital | Inclusion: follows: 1) Biopsy revealed malignant disease with ductal origin; and 2) additional breast MRI was conducted before surgery and deciphered by highly experienced radiologists.  Exclusion: Patients whose biopsy result was reported as other malignant breast diseases excluding ductal carcinoma, who developed other primary malignancies, and who underwent neoadjuvant chemotherapy or excisional biopsy before breast MRI were excluded. | 199 (all patients had a MRI) | NR | Mean age: 50.1 (9.4) | 1.5 T system  with a dedicated 4-channel breast coil. | - Treatment change |
| Obdeijn 2013 | Netherlands  2005 to 2010 | Non-concurrent retrospective cohort  MRI: April 2007 until July 2010  No-MRI: January 2005 to December 2006 | *All groups:*  Inclusion: patients with histopathologically proven breast cancer or DCIS who were eligible for breast-conserving surgery  *Patients with MRI group:*  Inclusion: underwent preoperative MRI of the breast.  Exclusion: contraindications for MRI, benignity of the lesion, metastatic disease, operation abroad, rejection of treatment, and neoadjuvant chemotherapy  *No-MRI group:*  Inclusion: patients who underwent breast-conserving surgery.  Exclusion: None | 38 (MRI: 11; no-MRI: 27) | Data was not reported for DCIS patients.  For all the 272 included lesions:   - Size mean (mm): MRI: 31.5, No-MRI: 21.4 - Microcalcification: NR | Data was not reported for DCIS patients.  For all the 272 included lesions:   - MRI: 54.0 (26-73) - No MRI: 55.2 (29-79) | 1.5-T system with a dedicated double breast coil. | - Positive margin status - Re- operation rate |
| Onega 2017 | United States | Concurrent retrospective cohort (Breast Cancer Surveillance Consortium (BCSC) Statistical Coordinating Center from 2010 to 2014) | Inclusion: Women diagnosed with an incident non-metastatic unilateral breast cancer (Stage 0-III) and who received their initial surgical treatment within 6 months of their cancer diagnosis.  Exclusion: Women who reported personal history of breast cancer. | 2437 (MRI: 354; No-MRI: 2083) | Data was not reported for DCIS patients.  For all the 13097 included patients:   - Size: 24.5% had less than 1cm - Microcalcification: NR | Data was not reported for DCIS patients.  For all the 13097 included patients: Median = 62 | NR | - Initial mastectomy - Initial BCS |
| Pettit 2009 | United States 2005 - 2008 | One-arm retrospective cohort from the Washington University/Barnes Jewish Hospital | Inclusion: patients with a diagnosis of Stage 0-III invasive breast cancer who underwent surgical treatment and MRI  Exclusion: Not stated, but they excluded some patients because of the following reasons: diagnosis of high risk lesion only (7); diagnosis was not adenocarcinoma (5); MRI performed to find primary for metastatic disease already diagnosed (16); prior history of treated ipsilateral breast cancer (18); diagnosis of inflammatory breast cancer (3); and MRI was not interpretable due to technical reasons (2). | 51 (all patients had a MRI) | NR | Data was not reported for DCIS patients.  For all the 441 included patients: 63.9% had between 40 and 60yo. | Siemens 1.5T Sonata or Espree magnetic resonance imaging unit. Patients were placed in the prone position and both breasts were imaged using a dedicated breast coil. | - Treatment change |
| Pilewskie 2013 | United States  2008 - 2010 | Concurrent prospective cohort from the Lynn Sage Comprehensive Breast Center | Inclusion: patients with core needle biopsy (CNB) of the breast showing pure DCIS, although <=1 mm of micro-invasion was allowed | 352 (MRI: 217; No-MRI: 135) | - Microcalcification: MRI: 160 (75.8 %), No-MRI: 330 (93.8 %) - Size mean cm (range): MRI: 2.1 (0.0, 10.0), No-MRI: 1.7 (0.0, 9.0), | - MRI: 53 (26 – 82) - No MRI: 60 (36-86) | NR | - Initial mastectomy - Initial BCS - Re- operation rate - Total mastectomy rate |
| Pilewskie 2014 | United States 1997 - 2010 | Concurrent retrospective cohort from the Memorial Sloan-Kettering Cancer Center (MSKCC) | Inclusion:  Patients with new diagnosis of DCIS, who underwent minimally invasive biopsy.  Exclusion: None | 2319 (MRI: 596; No-MRI: 1723) | NR | - MRI: 54.0 (26-73) - No MRI: 53.5 (25–85) | NR | - Positive margin status - Locoregional recurrence |
| Shin 2012 | Korea 2003 - 2005 | Concurrent retrospective cohort from the Seoul National University Hospital | Inclusion: “Women with invasive or in situ carcinoma with preoperative mammography and US, and breast MRI was conducted routinely starting in 2004”.  Exclusion: Those who had received neoadjuvant chemotherapy, and because preoperative breast imaging (US or MRI) was not done or because surgical pathology results were unavailable. | 87 (MRI: 62; no-MRI: 25) | Data was not reported for DCIS patients.  For all the 794 included patients:   - Microcalcification: NR - Size mean ± SD: Total: 3.05 ± 2.01, MRI: 3.17 ± 2.13, No-MRI: 2.28 ± 1.41 | Data was not reported for DCIS patients.  For all the 794 included patients:   - MRI: 46.3 - No MRI: 48.6 | A 1.5-T imager with dedicated double-breast coil was used. | - Re- operation rate |
| So 2018 | United States 2010 - 2013 | Concurrent retrospective cohort from one health establishment | Inclusion: women with pure DCIS treated by BCS  Exclusion: 76 were excluded because they had surgery elsewhere, and 44 because they underwent excisional biopsy instead of core needle biopsy. | 176 (MRI: 97; no-MRI: 79) | - Microcalcification: NR - Size mean ± SD: MRI: 1.5 ± 1.9, no-MRI 1.6 ± 2.6 | - MRI: 56.4 - No MRI: 63.6 | NR | - Re- operation rate |
| Vapiwala 2017 | United States 1992 - 2001 | Concurrent retrospective cohort from the Hospital of the University of Pennsylvania (HUP) | Inclusion: women with breast cancer treated by BCS + breast irradiation  Exclusion: None | 135 (MRI: 31; no-MRI: 104) | Data was not reported for DCIS patients.  For all the 755 included patients:   - Microcalcification: NR - Size (cm): MRI: <=2cm (51%), 2.1-5.0cm (7%); No-MRI: <=2cm (48%), 2.1-5.0cm (13%) | Data was not reported for DCIS patients.  For all the 755 included patients:   - MRI: 53 (25 - 85) - No MRI: 57 (27 – 89) | NR | - Locoregional recurrence |
| Vos 2015 | Netherlands 2011 - 2014 | Concurrent retrospective cohort, population-based Netherlands Cancer Registry (Eindhoven region) | Inclusion: Patients diagnosed with a new invasive breast cancer or DCIS.  Exclusion: male sex, neoadjuvant chemotherapy or hormone therapy, clinical or pathological tumour stage T4, distant metastasis at presentation, unknown pathological tumour stage or T0, and unknown type of surgery or unknown surgical margin status. | 614 (MRI: 136; no-MRI: 478) | NR | Data was not reported for DCIS patients.  For all the 6685 included patients:   - MRI: 46.9% had between 41 and 59yo - No MRI: 34.6% had between 41 and 59yo | Dynamic contrast-enhanced MRI | - Initial mastectomy - Initial BCS - Positive margin status - Re- operation rate - Total mastectomy rate |
| Yoon 2020 | Korea 2012 - 2016 | Retrospective cohort from the referral Gangneung Asan Hospital and recruited from the disease database | Inclusion: Women who were diagnosed consecutively with DCIS by using US-guided CNB only  Exclusion: Women who had concurrent invasive carcinoma, who had not undergone an operation, or who had a history of ipsilateral breast cancer were excluded. | Unmatched: 541 (MRI: 430; no-MRI: 111)  After propensity score matching: 212 (MRI: 106; no-MRI:106) | -Size (cm): 3.0 (2.4)  -Microcalcification: included lesions with calcification but percentage was not reported | Mean age: 53.5 (10) | 1.5-T  or 3.0-T  MR scanner and a dedicated 18-channel phased-array  breast coil with the patient in a  prone position. | - Initial mastectomy - Initial BCS - Positive margin status - Re- operation rate - Total mastectomy rate - Treatment change |

*Two studies reported in the same cohort of patients with DCIS, here we reported the publication describing the largest number of observations.

## Figure S1. Risk of bias of included studies

## 1A: Observational studies (ROBINS-I)

|  |  |  |  |  |  |  |  |  |  |
| --- | --- | --- | --- | --- | --- | --- | --- | --- | --- |
|  |  | Confounding | Selection of participants | Classification of intervention | Deviations from intended interventions | Missing data | Measurement of outcomes | Selection of reported result |  |
|  | Allen 2010 |  |  |  |  |  |  |  |  |
|  | Besharat 2018 |  |  |  |  |  |  |  |  |
|  | Davis 2012 |  |  |  |  |  |  |  |  |
|  | Duygulu 2012 |  |  |  |  |  |  |  |  |
|  | Hajaj 2017 |  |  |  |  |  |  |  |  |
|  | Hlubocky 2018 |  |  |  |  |  |  |  |  |
|  | Itakura 2011 |  |  |  |  |  |  |  |  |
|  | Keymeulen 2020 |  |  |  |  |  |  |  |  |
|  | Kropcho 2012 |  |  |  |  |  |  |  |  |
|  | Lam 2019 |  |  |  |  |  |  |  |  |
|  | Lamb 2020 |  |  |  |  |  |  |  |  |
|  | Lee 2016, 2020 |  |  |  |  |  |  |  |  |
|  | Obdeijn 2013 |  |  |  |  |  |  |  |  |
|  | Onega 2017 |  |  |  |  |  |  |  |  |
|  | Pettit 2009 |  |  |  |  |  |  |  |  |
|  | Pilewskie 2014 |  |  |  |  |  |  |  |  |
|  | Pilewskie 2013 |  |  |  |  |  |  |  |  |
|  | Shin 2012 |  |  |  |  |  |  |  |  |
|  | So 2018 |  |  |  |  |  |  |  |  |
|  | Vapiwala 2017 |  |  |  |  |  |  |  |  |
|  | Vos 2015 |  |  |  |  |  |  |  |  |
|  | Yoon 2020 |  |  |  |  |  |  |  |  |
|  |  |  |  |  |  |  |  |  |  |
|  | \|  \| Critical \|  \| Serious \|  \| Moderate \|  \| Low \| \| --- \| --- \| --- \| --- \| --- \| --- \| --- \| --- \| | | | | | | | |  |
|  |  | | | | | | | |  |

2B: Randomized clinical trial (Cochrane risk of bias tool)

|  |  |  |  |  |  |  |  |  |
| --- | --- | --- | --- | --- | --- | --- | --- | --- |
|  |  | Random sequence generation | Allocation concealment | Blinding of participants and personnel | Blinding of outcome assessment | Incomplete outcome data | Selective reporting |  |
|  | Balleyguier 2019 | 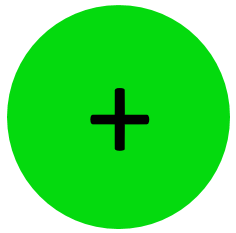 | 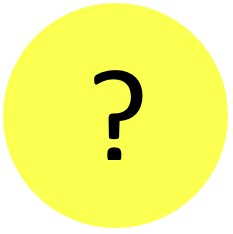 | 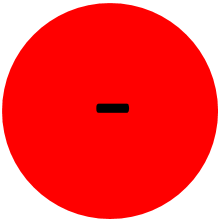 | 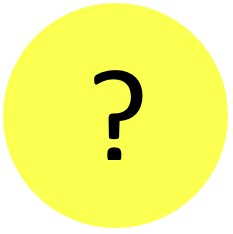 | 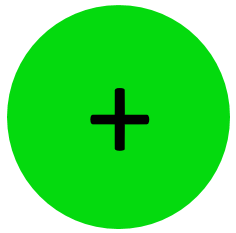 | 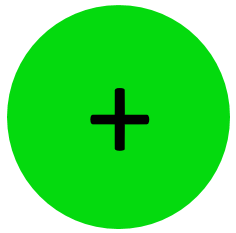 |  |
|  | Peters 2011 | 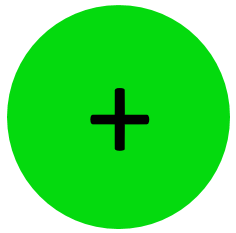 | 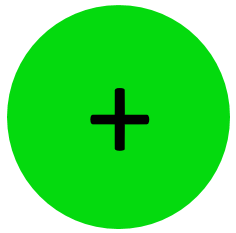 | 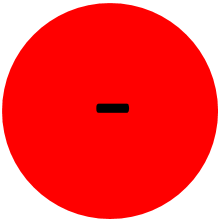 | 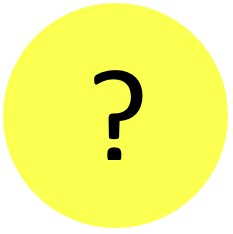 | 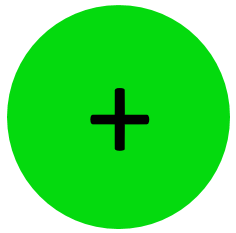 | 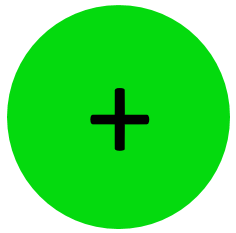 |  |
|  | Turnbull 2010 | 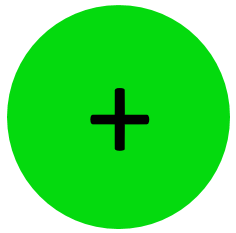 | 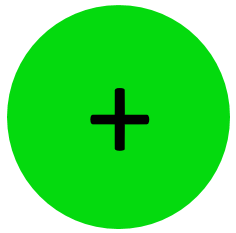 | 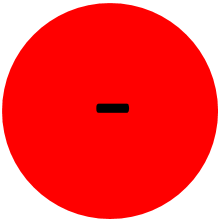 | 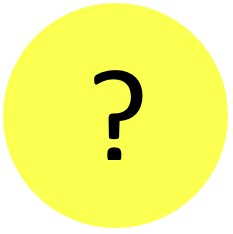 | 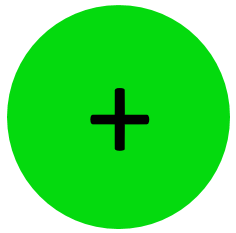 | 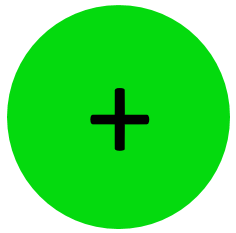 |  |
|  |  |  |  |  |  |  |  |  |
|  | \| 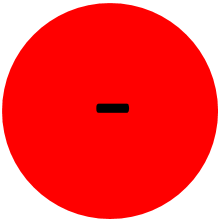 \| High \| 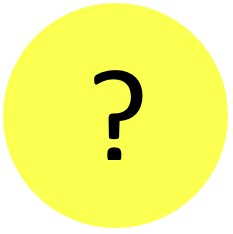 \| Unclear \| 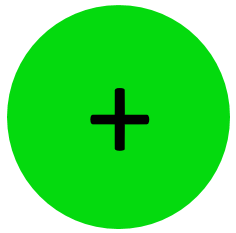 \| Low \| \| --- \| --- \| --- \| --- \| --- \| --- \| | | | | | | |  |
|  |  |  |  |  |  |  |  |  |

##
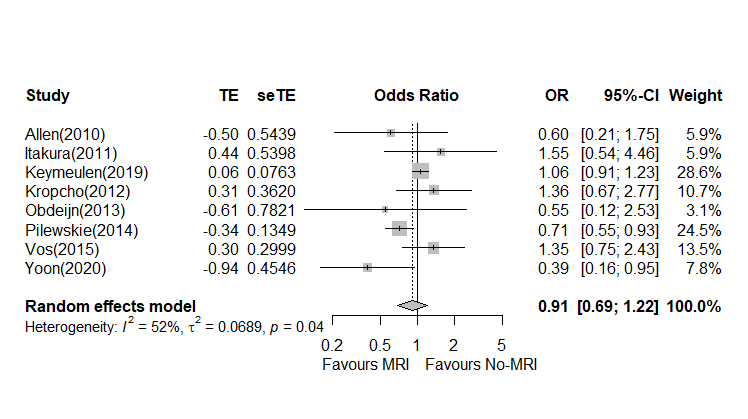

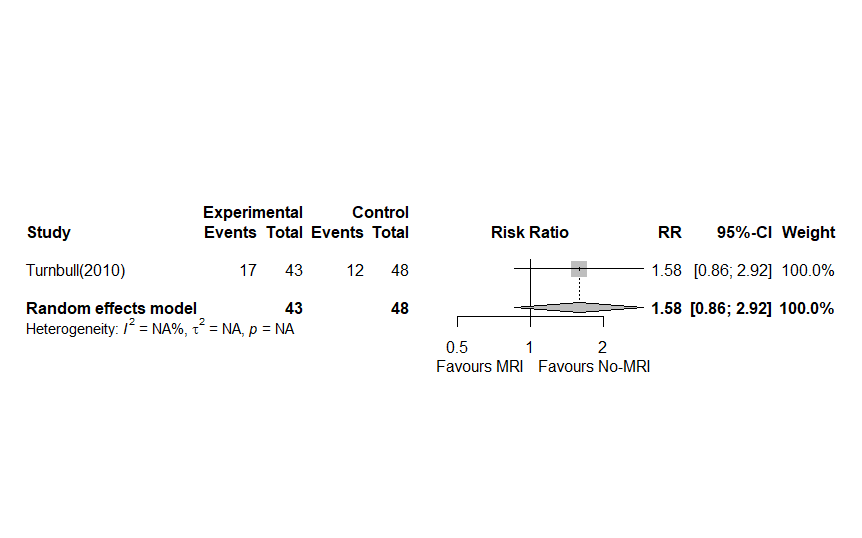
Figure S2. Proportion of positive margins

B

A

A: Randomized clinical trial; B: Cohort studies (prospective, and retrospective)

## Figure S3: Proportion treatment change triggered MRI


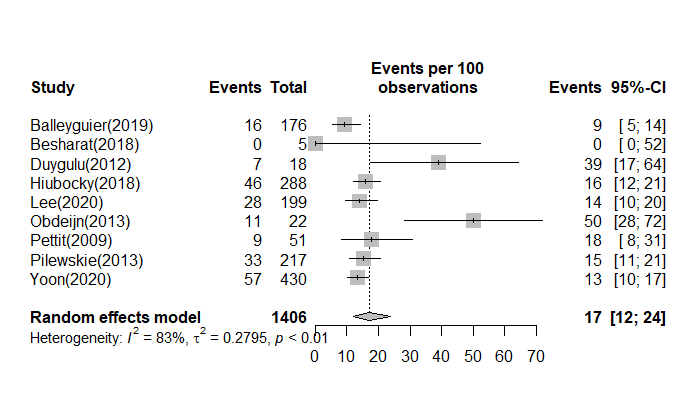


**Table S4. Subgroup analysis (observational studies)**

|  | **Initial BCS** | **n** | ***p value**** | **Re-operation** | **n** | ***p value**** | **Positive margins** | **n** | ***p value**** | **Total mastectomy** | **n** | ***p value**** |
| --- | --- | --- | --- | --- | --- | --- | --- | --- | --- | --- | --- | --- |
| Patients with Microcalcification |  |  |  |  |  |  |  |  |  |  |  |  |
| Not Reported | 0.46 (0.23 to 0.89) | 9 | 0.651 | 0.68 (0.43 to 1.07) | 10 | 0.365 | 0.91 (0.69 to 1.22) | 8 | --- | 1.58 (0.93 to 2.69) | 7 | 0.912 |
| Reported | 0.55 (0.36 to 0.85) | 2 |  | 0.88 (0.63 to 1.21) | 2 |  | --- | --- |  | 1.52 (0.95 to 2.42) | 1 |  |
| Risk of Bias |  |  |  |  |  |  |  |  |  |  |  |  |
| High | 0.49 (0.29 to 0.82) | 11 | ---- | 0.68 (0.47 to 0.98) | 11 | 0.067 | 0.91 (0.69 to 1.22) | 8 | ---- | 1.58 (1.00 to 2.48) | 8 | --- |
| Low | ---- | ---- |  | 1.77 (0.68 to 4.60) | 1 |  | ---- | --- |  | ---- | -- |  |
| Design |  |  |  |  |  |  |  |  |  |  |  |  |
| Retrospective | 0.47 (0.26 to 0.83) | 10 | 0.259 | 0.71 (0.47 to 1.07) | 11 | 0.928 | 0.92 (0.69 to 1.22) | 8 | ---- | 1.58 (0.93 to 2.69) | 7 | 0.912 |
| Prospective | 0.71 (0.45 to 1.14) | 1 |  | 0.73 (0.40 to 1.33) | 1 |  | ---- | --- |  | 1.52 (0.95 to 2.42) | 1 |  |
| Analysis |  |  |  |  |  |  |  |  |  |  |  |  |
| Crude | 0.48 (0.25 to 0.90) | 9 | 0.918 | 0.66 (0.45 to 0.99) | 9 | 0.499 | 0.93 (0.74 to 1.17) | 6 | 0.751 | 1.51 (0.89 to 2.57) | 6 | 0.828 |
| Adjusted | 0.51 (0.19 to 1.34) | 2 |  | 0.96 (0.36 to 2.61) | 3 |  | 0.77 (0.23 to 2.56) | 2 |  | 1.74 (0.53 to 5.68) | 2 |  |
| Publication year |  |  |  |  |  |  |  |  |  |  |  |  |
| 2015 to 2020 | 0.42 (0.22 to 0.83) | 7 | 0.494 | 0.79 (0.45 to 1.38) | 7 | 0.586 | 0.89 (0.46 to 1.76) | 3 | 0.832 | 1.60 (0.80 to 3.17) | 4 | 0.935 |
| Before 2015 | 0.63 (0.26 to 1.53) | 4 |  | 0.65 (0.45 to 0.95) | 5 |  | 0.83 (0.60 to 1.13) | 5 |  | 1.53 (0.76 to 3.09) | 4 |  |
| *Test for subgroup differences (random effects model)  **Only two observational studies reported the percentage of patients with microcalcified lesions. Thus, the analysis compared reported vs not reported lesions. | | | | | | | | | | | | |

**Table S5. Evidence Profile**

| **Certainty assessment** | | | | | | | **№ of patients** | | **Effect** | | **Certainty** | **Importance** |
| --- | --- | --- | --- | --- | --- | --- | --- | --- | --- | --- | --- | --- |
| **№ of studies** | **Study design** | **Risk of bias** | **Inconsistency** | **Indirectness** | **Imprecision** | **Other considerations** | **additional MRI** | **no additional MRI** | **Relative (95% CI)** | **Absolute (95% CI)** |  |  |
| **Initial breast conservative surgery - RCT** | | | | | | | | | | | | |
| 2 ^1,2^ | randomised trials | serious ^a,b^ | not serious | serious ^c^ | not serious | none | 187/215 (87.0%) | 193/214 (90.2%) | **RR 0.95** (0.90 to 1.00) | **45 fewer per 1,000** (from 90 fewer to 0 fewer) | ⨁⨁⨁◯ MODERATE | CRITICAL |
| **Initial breast conservative surgery - Cohorts** | | | | | | | | | | | | |
| 11 ^3,4,5,6,7,8,9, 19, 20, 22, 23^ | observational studies | very serious ^d,e^ | serious ^f^ | serious ^c^ | not serious | none ^g^ | 2028/4089 (50.0%) | 8345/11032 (75.6%) | **OR 0.49** (0.29 to 0.82) | **153 fewer per 1,000** (from 283 fewer to 38 fewer) | ⨁◯◯◯ VERY LOW | CRITICAL |
| **Proportion of positive margins - RCT** | | | | | | | | | | | | |
| 1 ^10^ | randomised trials | serious ^a,b,h^ | not serious | not serious | very serious ^i^ | none | 17/43 (39.5%) | 12/48 (25.0%) | **RR 1.58** (0.85 to 2.92) | **145 more per 1,000** (from 38 fewer to 480 more) | ⨁◯◯◯ VERY LOW | CRITICAL |
| **Proportion of positive margins - Cohorts** | | | | | | | | | | | | |
| 8 ^3,6,9, 11,12, 14, 20, 22^ | observational studies | very serious ^d,e,h^ | serious ^e^ | not serious | not serious ^j^ | none | 413/2113 (18.6%) | 1438/7013 (20.5%) | **OR 0.91**  (0.69 to 1.22) | **15 fewer per 1,000** (from 54 fewer to 34 more) | ⨁◯◯◯ VERY LOW | CRITICAL |
| **Re-operation rate - RCT** | | | | | | | | | | | | |
| 2 ^1,2^ | randomised trials | serious ^a,b^ | not serious | not serious | very serious ^i^ | none | 50/185 (27.0%) | 57/192 (29.7%) | **RR 1.02** (0.56 to 1.84) | **6 more per 1,000** (from 131 fewer to 249 more) | ⨁◯◯◯ VERY LOW | CRITICAL |
| **Re-operation rate - Cohorts** | | | | | | | | | | | | |
| 3 ^3,13, 22^ | observational studies | serious ^l^ | not serious | not serious | serious ^i^ | none | 49/280 (17.5%) | 95/576 (16.4%) | **OR 0.96** (0.36 to 2.61) ^j^ | **6 fewer per 1,000** (from 99 fewer to 175 more) | ⨁⨁◯◯ LOW | CRITICAL |
| **Total mastectomy rate – RCT** | | | | | | | | | | | | |
| 2 ^1,2^ | randomised trials | serious ^a,b^ | not serious | not serious | very serious ^i^ | none | 46/215 (21.4%) | 50/214 (23.4%) | **RR 0.91** (0.65 to 1.27) | **21 fewer per 1,000** (from 82 fewer to 63 more) | ⨁◯◯◯ VERY LOW | CRITICAL |
| **Total mastectomy rate - Cohorts** | | | | | | | | | | | | |
| 8 ^3,4,8,9,12,19,20, 22^ | observational studies | very serious ^d,e^ | serious ^f^ | not serious | not serious ^j^ | none | 1607/3391 (47.4%) | 2640/8892 (29.7%) | **OR 1.58** (1.00 to 2.48) | **103 more per 1,000** (from 0 more to 215 more) | ⨁◯◯◯ VERY LOW | CRITICAL |
| **Disease free survival (inferred form locoregional recurrence) - Cohorts** | | | | | | | | | | | | |
| 1 ^11^ | observational studies | serious ^m^ | not serious | serious ^n.o^ | serious ^p^ | none | 37/581 (6.4%) | 139/1631 (8.5%) | **HR 1.18** (0.79 to 1.76) | **15 more per 1,000** (from 17 fewer to 60 more) | ⨁⨁◯◯ LOW | CRITICAL |
| **Treatment change - Cohort** | | | | | | | | | | | | |
| 10 ^2,4,14,15,16,17,18, 21, 22, 24^ | observational studies | serious ^q^ | serious ^f^ | not serious | not serious | none | The pooled proportion of treatment change was 16% (95% CI 12% to 22%; I^2^ 79%). (N= 1560). | | | | ⨁⨁◯◯ LOW | CRITICAL |

**CI:** Confidence interval; **RR:** Risk ratio; **OR:** Odds ratio; **HR:** Hazard Ratio

#### Explanations

a. The intervention (preoperative MRI) was not feasible to be blinded which originated high risk of performance bias, potentially influencing surgeons’ treatment plans.

b. There was also a potential risk of imbalance of prognostic factors, due to including results from very small subgroup of participants in some RCTs.

c. Initial BCS was considered an intermediate outcome, as women could have received re-excision or a mastectomy depending of the presence of positive margins in the excised specimen.

d. In some cohort studies the comparison was between arms over different periods of time (secular bias).

e. Most observational studies reported unadjusted estimates.

f. Potentially important and unexplained heterogeneity across studies.

g. Although there is an observed large effect, there is a very serious concerns of risk of bias and the estimate is imprecise, thus, we did not upgrade the certainty of evidence.

h. The definition of positive margins was variable across clinical centers potentially introducing misclassification bias.

i. There anticipated absolute effects associated to the intervention goes from potential benefit to potential harm.

j. There is imprecision of the anticipated absolute effects with the intervention, but it is likely due to heterogeneity across studies.

k. Only estimates from studies reporting adjusted ORs are included as the results were different from those unadjusted.

l. Both studies reported adjusted estimates, although one study did not include tumour size as a pre-defined confounding variable in the analysis. Additionally, there was no information about the time the MRI exam was requested.

m. Only patients that received breast conservative surgery were included. Potential selection bias as those with more aggressive treatments after MRI were not included. Potential over adjustment in the multivariate analysis as positive margins and number of excisions may be in the casual pathway to disease recurrence.

n. A proportion of patients had breast MRI performed after lumpectomy or at re-excision stage.

o. Serious indirectness as locoregional recurrence is considered a surrogate of disease-free survival.

p. The absolute effect of the intervention ranged from significant benefit to significant harm.

q. Decision to request breast MRI (after mammography and ultrasound) might be associated to the decision to change the initial plan, independently of MRI results

#### References

1. Peters NH, van Esser S,van den Bosch MA,Storm RK,Plaisier PW,van Dalen T,Diepstraten SC,Weits T,Westenend PJ,Stapper G,Fernandez-Gallardo MA,Borel Rinkes IH,van Hillegersberg R,Mali WP,Peeters PH.. Preoperative MRI and surgical management in patients with nonpalpable breast cancer: the MONET - randomised controlled trial. Eur J Cancer; 2011.

2. Balleyguier C, Dunant A,Ceugnart L,Kandel M1 Chauvet MP,Chérel P,et al.. Preoperative Breast Magnetic Resonance Imaging in Women With Local Ductal Carcinoma in Situ to Optimize Surgical Outcomes: Results From the Randomized Phase III Trial IRCIS.. J Clin Oncol.; 2019.

3. Vos, E. L., Voogd, A. C., Verhoef, C., Siesling, S., Obdeijn, I. M., Koppert, L. B.. Benefits of preoperative MRI in breast cancer surgery studied in a large population-based cancer registry. Br J Surg; Dec 2015.

4. Pilewskie M, Kennedy C,Shappell C,Helenowski I,Scholtens D,Hansen N,Bethke K,Jeruss J., Karstaedt, P., Khan, S. A.. Effect of MRI on the management of ductal carcinoma in situ of the breast. Ann Surg Oncol; 2013.

5. Onega T, Weiss JE,Goodrich ME,Zhu W,DeMartini WB,Kerlikowske K,Ozanne E,Tosteson A. N. A., Henderson, L. M., Buist, D. S. M., Wernli, K. J., Herschorn, S. D., Hotaling, E., O'Donoghue, C., Hubbard, R.. Relationship between preoperative breast MRI and surgical treatment of non-metastatic breast cancer. J Surg Oncol; 2017.

6. Itakura K, Lessing J,Sakata T,Heinzerling A,Vriens E,Wisner D,Alvarado M,Esserman L,Ewing C,Hylton N,Hwang ES.. The impact of preoperative magnetic resonance imaging on surgical treatment and outcomes for ductal carcinoma in situ. Clin Breast Cancer; 2011.

7. Hajaj, M., Karim, A., Pascaline, S., Noor, L., Patel, S., Dakka, M.. Impact of MRI on high grade Ductal Carcinoma Insitu (HG DCIS) management, are we using the full scope of MRI?. Eur J Radiol; Oct 2017.

8. Davis, K. L., Barth, R. J.,Jr., Gui, J., Dann, E., Eisenberg, B., Rosenkranz, K.. Use of MRI in preoperative planning for women with newly diagnosed DCIS: risk or benefit?. Ann Surg Oncol; Oct 2012.

9. Allen, L. R., Lago-Toro, C. E., Hughes, J. H., Careaga, E., Brown, A. T., Chernick, M., Barrio, A. V., Frazier, T. G.. Is there a role for MRI in the preoperative assessment of patients with DCIS?. Ann Surg Oncol; Sep 2010.

10. Turnbull, L. W., Brown, S. R., Olivier, C., Harvey, I., Brown, J., Drew, P., Hanby, A., Manca, A., Napp, V., Sculpher, M., Walker, L. G., Walker, S.. Multicentre randomised controlled trial examining the cost-effectiveness of contrast-enhanced high field magnetic resonance imaging in women with primary breast cancer scheduled for wide local excision (COMICE). Health Technol Assess; Jan 2010.

11. Pilewskie, M., Olcese, C., Eaton, A., Patil, S., Morris, E., Morrow, M., Van Zee, K. J.. Perioperative breast MRI is not associated with lower locoregional recurrence rates in DCIS patients treated with or without radiation. Ann Surg Oncol; May 2014.

12. Kropcho, L. C., Steen, S. T., Chung, A. P., Sim, M. S., Kirsch, D. L., Giuliano, A. E.. Preoperative breast MRI in the surgical treatment of ductal carcinoma in situ. Breast J; Mar-Apr 2012.

13. So, A., De La Cruz, L. M., Williams, A. D., Bahng, J., Liao, G., McDonald, E. S., Fisher, C. S., Czerniecki, B. J., Sataloff, D., Tchou, J.. The impact of preoperative magnetic resonance imaging and lumpectomy cavity shavings on re-excision rate in pure ductal carcinoma in situ-A single institution's experience. J Surg Oncol; Mar 2018.

14. Obdeijn IM, Tilanus-Linthorst MM,Spronk S,van Deurzen CH,de Monye C,Hunink MG,Menke MB.. Preoperative breast MRI can reduce the rate of tumor-positive resection margins and reoperations in patients undergoing breast-conserving surgery. AJR Am J Roentgenol; 2013.

15. Pettit, K., Swatske, M. E., Gao, F., Salavaggione, L., Gillanders, W. E., Aft, R. L., Monsees, B. S., Eberlein, T. J., Margenthaler, J. A.. The impact of breast MRI on surgical decision-making: are patients at risk for mastectomy?. J Surg Oncol; Dec 1 2009.

16. Lee, J., Jung, J. H., Kim, W. W., Hwang, S. O., Kim, H. J., Park, J. Y., Chae, Y. S., Yang, J. D., Park, H. Y.. The role of preoperative breast magnetic resonance (MR) imaging for surgical decision in patients with triple-negative breast cancer. J Surg Oncol; Jan 2016.

17. Hlubocky, J., Bhavnagri, S., Swinford, A., Mitri, C., Rebner, M., Pai, V.. Does the use of pretreatment MRI change the management of patients with newly diagnosed breast cancer?. Breast J; Nov 5 2017.

18. Duygulu G, Oktay A,Bilgen IG,Kapkaç M,Zekioğlu O.. The role of breast MRI in planning the surgical treatment of breast cancer. Diagn Interv Radiol; 2012.

19. Lam DL, Smith J, Partridge SC, et al. The Impact of Preoperative Breast MRI on Surgical Management of Women with Newly Diagnosed Ductal Carcinoma In Situ. Academic radiology 2020.

20. Keymeulen K, Geurts SME, Lobbes MBI, et al. Population-based study of the effect of preoperative breast MRI on the surgical management of ductal carcinoma in situ. The British journal of surgery 2019

21 Besharat Sara KM, Julaee Azadeh, Malekpour Nasser. Influence of preoperative magnetic resonance imaging in surgical planning for breast cancer. Int J Cancer Manag 2018

22 Yoon GY, Choi WJ, Kim HH, Cha JH, Shin HJ, Chae EY. Surgical Outcomes for Ductal Carcinoma in Situ: Impact of Preoperative MRI. Radiology. 2020

23 Lamb L, Oseni T, Lehman CD, Bahl M. Pre-operative MRI in patients with ductal carcinoma in situ: Is MRI useful for identifying additional disease?. European Journal Radiology 2020.

24 Lee J, Jung JH, Kim WW, Park CS, Lee RK, Kim HJ, Kim WH, Park HY. Efficacy of breast MRI for surgical decision in patients with breast cancer: ductal carcinoma in situ versus invasive ductal carcinoma. BMC Cancer. 2020
